# 

**Published:** 1816-08

**Authors:** 


### MONTHLY CATALOGUE OF MEDICAL BOOKS.

In a few days will be published, the Translation of the concluding Part of Orfila's General System of Animal, Mineral, and Vegetable Poisons.

Speedily will be published, in one small octavo volume, a correct Translation of Blumenbach's Work on the Variety of the Human Species.

In a short time will be published, a large Print, representing an Anterior and Posterior View of the Leg and Foot, shewing the principal Blood Vessels, Nerves, and Muscles of this Part of the Human Body; coloured according to Nature.

Shortly will be published, a Translation of Professor Majendi's new Work on Physiology, with occasional Notes.

In a few weeks will be published, the First Volume of a New and Complete System of Anatomy, translated from the French of Bichat's Anatomie Generale et Descriptive; the whole will be completed in three octavo volumes, accompanied by plates.

In a few weeks will be published, a Manual of Materia Medica; designed principally for the use of those who are preparing for their Examination at Apothecaries' Hall.

---

An Answer to Dr. Kinglake shewing the Danger of the Cooling Treatment of the Gout. By John Ring, Member of the Royal College of Surgeons of London and Paris.

Medico-Chirurgical Transactions, vol. 7, part i.

A Treatise on the Nature and Cure of Gout; comprehending a General View of a Morbid State of the Digestive Organs, and of Regimen, with some Observations on Rheumatism. By Charles Scudamore, Member of the College of Physicians, of the Medical and Chirurgical Society in London, &c. 8vo.

Practical Observations on the Diseases of the Urinary Organs, particularly those of the Bladder, Prostate Gland, and Urethra, illustrated by Cases and Engravings. By John Howship, Surgeon, &c. 8vo.

An Enquiry into the History and Nature of the Disease produced in the Human Constitution by the Use of Mercury, with Observations on its Connection with the Lues Venerea. By Andrew Mathias, Surgeon-Extraordinary to the Queen, &c. 8vo.

---

### *NOTICES TO CORRESPONDENTS.*

*We are much flattered by* Mr. Litteton's *early answer. Having taken the opinion of a most experienced practical Oculist, we are so far confirmed on the perspicuousness of* Mr. L.'s *MS. that we shall at present, at least, defer the plates till we see how far they may be necessary to illustrate the remaining part of his communication.*

Mr. Ward's *communication shall be duly attended to. As he has given us no other address than London, he will see the propriety of a little caution on our part. We hope in a few days to procure* Mr. Kite's *Essay, after which we shall think it our duty to treat our other Correspondent according to his merits. If Mr. Ward will favor us with his address, we will take the liberty of applying to him for Mr. Kite.*

*We have received from the authors or publishers several books for our Critical department, all which shall be noticed in their turn.*

*The favors of* W. S. Clark, Esq. E. H., *and others, shall be duly attended to.*

